# Behind the closed doors of mentalizing. A commentary on “Another step closer to measuring the ghosts in the nursery: preliminary validation of the Trauma Reflective Functioning Scale”

**DOI:** 10.3389/fpsyg.2015.00380

**Published:** 2015-04-01

**Authors:** Adriano Schimmenti

**Affiliations:** Faculty of Human and Social Sciences, UKE – Kore University of EnnaEnna, Italy

**Keywords:** ghosts in the nursery, dissociation, mentalization, trauma, attachment, child abuse/neglect

In his *Clinical Diary*, Ferenczi (1932/1988) suggested that an organizing life instinct allows the individual to survive child abuse. He called this instinct Orpha, and described it as a guardian angel who anesthetizes “the consciousness and sensitivity against sensations as they become unbearable” (p. 9). Ferenczi argued that a fragmentation however occurs in personality as a consequence of the abuse: the personality is split into a “capable part” as a “regulated mechanism” dealing with daily life and activities, secret parts that struggle in despair because they experience “the fire of suffering,” and another part containing “this suffering itself as a separate mass of affect, without contents and unconscious, the remains of the actual person” (p. 10).

Ferenczi's concept of Orpha tends to correspond to our current understanding of dissociation. In fact, child abuse and neglect (CA&N) in the context of attachment relationship can generate a severe impairment in the individual's ability to integrate mental states and their related affective contents into a consistent structure of meaning (Allen, 2013). The psychological cost of dissociation is high: dissociation may involve either a loss of continuity in subjective experience, and/or an inability to access information or control mental functions, and/or a sense of experiential disconnectedness (Cardeña and Carlson, 2011). How do these considerations relate to the “ghosts in the nursery” (Fraiberg et al., 1975), the haunting internal presences that lead parents to re-enact their own traumatic past by victimizing their child?

In a recent study, Ensink et al. (2014) investigated how mentalization (measured as reflective functioning, RF) was related to investment in pregnancy and couple functioning among 100 pregnant women who suffered from CA&N. The researchers analyzed the Adult Attachment Interview protocols (AAI) of these women, and applied distinct RF scores to the relationship with attachment figures (RF-G) and to the traumatic experiences (RF-T). Ensink and colleagues found that RF-T was significantly lower than RF-G among the participants, thus they suggested that women with histories of CA&N do not manifest a generic inhibition of reflectiveness, but a collapse of mentalization specific to trauma. This finding is highly consistent with Ferenczi's early conceptualization of a dissociative part of personality deriving from CA&N and dealing with traumatic affects; it is also consistent with the current theory of structural dissociation (Van der Hart et al., 2006), which postulates a separation between the apparently normal part and the emotional part of personality in complex post-traumatic stress disorders.

Further attention deserves another interesting finding in the study by Ensink and colleagues. After running multiple regression analyses to identify the best predictors of engagement in pregnancy and quality of couple functioning among the women in the sample, these researchers found that RF-T was the only significant predictor in the regression models. However, they only entered RF-T scores, RF-G scores, unresolved vs. non-unresolved attachment, and secure vs. insecure attachment as potential predictors in the regression analyses. They did not control for other variables that were already available in their study and could account for the study findings, namely CA&N scores and AAI Unresolved trauma scores. Despite this limitation, Ensink et al. argued that “it is not the experience of trauma *per se*, but *the absence of mentalization regarding trauma* that is associated with difficulties in close relationships and in making the transition to parenthood” (original italics).

Notably, CA&N can hinder the development of both mentalization and affect regulation abilities (Allen, 2013). In fact, it has been suggested that lack of mentalization and affect dysregulation can be considered as complementary dimensions of the dissociative process deriving from CA&N (Schimmenti and Caretti, 2014); moreover, it has been proposed that AAI indicators of unresolved trauma reflect the severity of dissociative process resulting from CA&N (Liotti, 2004). These latter considerations could be particularly relevant for understanding the ghosts in the nursery. As Fraiberg et al. (1975) observed in their study, “memory for the events of childhood abuse, tyranny, and desertion was available in explicit and chilling detail. *What was not remembered was the associated affective experience*” (p. 419, original italics). On a theoretical level, lack of mentalization about traumatic experiences could be a developmental effect of CA&N and subsequent dissociation, rather than a specific cause of the maternal difficulties; on a research level, RF-T scores should prove to predict maternal attitudes toward pregnancy and couple functioning over and above trauma scores, before attributing such a predominant position to (lack of) mentalization in the intergenerational transmission of trauma.

Without any doubt, the research by Elsink and colleagues further supported the validity of the “ghosts in the nursery” construct. However, behind the closed doors of mentalizing, the fire of suffering and the dissociated mass of traumatic affects may continue to howl, unseen and unrecognized, around the child.

## Conflict of interest statement

The author declares that the research was conducted in the absence of any commercial or financial relationships that could be construed as a potential conflict of interest.
